# Could posture reflect welfare state? A study using geometric morphometrics in riding school horses

**DOI:** 10.1371/journal.pone.0211852

**Published:** 2019-02-05

**Authors:** Emilie Sénèque, Clémence Lesimple, Stéphane Morisset, Martine Hausberger

**Affiliations:** 1 Université de Rennes, UMR 6552 CNRS Ethologie Animale et Humaine,CNRS, Université de Caen-Normandie, Rennes, France; 2 Independent biostatistician, France; 3 CNRS, UMR 6552 Ethologie animale et humaine, Université de Rennes, Université de Caen-Normandie, Caen, France; University of Illinois, UNITED STATES

## Abstract

Despite the fact that animal posture is known to reflect emotional state, the presence of chronic postures associated with poor welfare has not been investigated with an objective tool for measuring, quantifying and comparing postures. The use of morphometric geometrics (GM) to describe horse posture (profile of the dorsum) has shown to be an effective method of distinguishing populations that are known to differ in terms of welfare states. Here we investigated photographs of 85 riding school horses differing in terms of welfare state, in order to determine if a specific posture (modelled by GM) is associated with altered welfare. The welfare state was estimated with the prevalence of stereotypic or abnormal repetitive behaviours, depressed-like posture and the ear positions. ANOVA results show that horses with stereotypic or abnormal behaviour, and to a lesser degree horses with depressed-like postures, tend to have a flatter, or even hollow, dorsal profile, especially at the neck and croup levels. These altered profiles could represent an additional indicator of poor welfare, easy to use in the field or by owners.

## Introduction

The early descriptions by Darwin in 1872 [1] of the expression of emotions in man and animals indicated that postures and emotions are clearly interrelated: changes in agonistic postures accompanying aggressive encounters in birds [2–3], dogs [4] and cats [1]; emotional responses to challenging situations in ungulates [5–7], fear or anxiety in dogs, mice [8] and other animals [9–10]. Although these postures are well known, precise quantitative evaluation is often lacking, while this would be of great interest for comparing situations or emotional states. Thus, postures are often described either subjectively [11–14], or quantitatively by measuring the positions of only some parts of the body, e.g. ears, neck, tail or trunk (*e*.*g*. [5; 6; 8]), or by measuring the general position of the body in space [14; 15]. A few studies have used measures of one or several angles between different parts of the body [3; 16].

The measurement of postures is especially important when considering animal welfare issues. It has been proposed that the welfare state results from the subjective perception of the situation by the individual [17]. The repetition of stressful situations over time may lead to chronic states and one can wonder whether this repetition may also lead to the repetition of the same associated postures and whether these may also become chronic. This effect of the “mental” state of the animal may thus compound health and physical constraints [18].

A few recent studies, based on the use of geometric morphometrics to quantify and compare body postures, suggest that this is indeed the case. Thus, the backs of pigs reared in social isolation were rounder (*i*.*e*. more tensed) than those of pigs reared with conspecifics [19]. Horses living in more natural conditions and also subjected to less constraining riding techniques have been shown to have more “optimistic” cognitive biases [20], and an overall “rounder” body posture than horses living in restricted conditions [21, 22]. Moreover it was shown that the roundness of the neck reflected the state of the horse’s back [22].

In the present study, we hypothesized that horses with welfare issues have chronic body postures associated with particular expressions of welfare problems they encounter. Horses are well known for responding to restrictive conditions by developing abnormal behaviours such as stereotypies (*e*.*g*. [23]), expressing “depressed” postures [24–27] or higher emotional states [28]. Several studies emphasize the role of the lack of roughage in the diet [29–31], or the spatial [29; 32–33] and social [34–35] restrictions on the emergence of such behaviours, but the riding techniques may also have important chronic consequences beyond work periods [36–38] (see overview in [39]).

Stress, fear, discomfort or pain produce contractions of the back muscles which impair the biomechanics of the dorsal body outline (*i*.*e*. back and neck) and result in deterioration of the musculoskeletal structures [40–41]. When repeated, the physical and psychological tensions could lead to chronic internal states reflected in the postures adopted. Previous studies on the posture of pigs and horses [21] have focused on the dorsal body outline of the body shape because of the predisposition of this structure to contractions and injuries. For example, an arched back shape is regarded as one element of some pain assessment in domestic animals (*e*.*g*. sheep [42–43], cattle [44–45], pig [46] and horse [47–49]).

In horses, back problems are a common cause of clinical presentation [49–50]. In a study on 2956 horses from leisure, stud farm, competition or riding schools, Visser et al. [51] noticed that 31% showed weak to strong responses to back palpation (indicating an intention to avoid pain); riding school horses were twice as often affected with back pain than other working horses. These observations were in accordance with those of Fureix et al. [52] and Lesimple et al. [22; 38; 53], who showed that 55 to 74% of riding school horses were severely affected by back disorders. Several studies highlight that the type and localization of vertebral disorders or soft tissue injuries differ according to diverse parameters related to riding: riding techniques, equipment (*e*.*g*. type of bit and saddle) [41; 54–55] or discipline [37; 39; 56].

Despite their wide prevalence, the presence and intensity of back problems are often underestimated by horse owners [53; 55; 57]. These features are associated with a flat or hollow neck and/or back, and a stiff back [40; 58]. However the presence, exact location and origin of back disorders are complicated to diagnose, even for trained experts, especially because of the thickness of soft tissues (*i*.*e*. adipose and muscle tissues) which limits radiographic and scintigraphic examination [49–50; 59; 60]. If postural alterations reflect particular problems in horse management, it would be important to characterize them by accurate postural measurements. In horses, kinematic studies exist, but they are often associated with invasive procedures (surgical implantation of markers) and they require that the animals are placed in standardized conditions which are unnatural [46; 61–63]. They also produce a plethora of data which is not easy to analyse, especially combined with other data in multivariate analysis.

In the present study therefore, we used the recently developed method of posture analysis based on geometric morphometrics (GM) applied to the back, neck and head of horses [21; 25] and pigs [19; 46]. GM is based on the analysis of coordinates in space, and is highly sensitive to small changes in form. We restricted our investigation to the dorsal outline of the animal excluding the position of the limbs which would introduce too much noise into the analysis. We also applied techniques developed specifically for this type of study [64], using an increased number of landmarks, sliding semi-landmarks (*i*.*e*. outline analysis method) and by eliminating the balance movements of the neck (in order to focus on the neck shape). These new methodological approaches allow us to define more precisely the dorsal outline, while reducing the ‘noise’ due to the movement of the neck.

## Materials and methods

Experiments complied with current French laws (Centre National de la Recherche Scientifique) related to animal experimentation and were in accordance with the European directive 86/609/CEE. No license/permit/institutional ethical approval was needed. Animal subjects were not exposed to distressing conditions during the study. Animal husbandry and care were under the management of riding school staff. Riding schools' managements authorized the experimenters to conduct their research. This experiment involved only horses in the “field” (no laboratory animals).

### Horses (see also Lesimple et al., [38])

Eighty-five horses were observed between October 2010 and May 2011 in 11 riding schools (N = 85 horses, 50 geldings and 35 mares, 1 to 13 horses per riding school) all over France. This sample constituted a subsection of a larger study [38]. All horses had water ad libitum, and most of them (87.1%) had one to three concentrate meals, and hay from one meal to *ad libitum*. The horses worked in riding lessons involving children and teenagers, with at least one free day per week. They were only used for teaching, with riders from beginner to intermediate levels. None of the horses observed was lame.

#### Type of equids and proportion

Official identification documents record the **sex**, **age** and breed of horses. Amongst the 85 animals, 37 were unregistered, and the rest was diverse (9 breeds) so we could not use the breed parameter. Given that other studies (e.g. [38; 65]) highlighted differences between **types of equids** (e.g. pony / horses, “warmbloods”/ “coldbloods”) the animals were divided into two “classical” official types: pony (<1.48m high at the withers, International Federation for Equestrian Sport) or horse (>1.48m high at the withers) in the analysis.

Using the parameters described in Chabchoub et al. [66], the animals were classified into 3 categories, based on their **proportions**: dolichomorphic (length > height at withers, e.g.: Thoroughbred, Purebred Arab), mesomorphic (length = height at withers, ex: French saddlebred) and brachymorphic (length < height at withers: e.g. Merens horses).

#### Management conditions

Horses were mostly (i.e. > 50% time) housed in straw bedded individual stalls (91.8%) or in group pastures (8.2%).

Some studies underline the importance of different management factors for horse welfare: feeding (*e*.*g*. [30; 31]), housing (*e*.*g*. [67]), social conditions (*e*.*g*. [68]) and working conditions(*e*.*g* [36–39]. Lesimple et al [38] have shown that all these parameters are influential but that their degree of influence differed. Therefore, for each individual, the following management parameters were recorded:

- the **number of hay meals,** giving information on the temporal distribution of feeding.
- **the percentage of time spent in paddock**,
- When they had access to a paddock, whether **alone or in group**.
- the **number of visible conspecifics when in stall**
- the **time spent working per week**, as reported in the official working document of the riding schools.

### Welfare indicators (see also Lesimple et al. [38])

The same behavioural and postural welfare indicators were used as in Lesimple et al [38] (recorded by one single observer, CL):

#### Behavioural measures: Stereotypic/abnormal repetitive behaviours

We recorded “stereotypic behaviours” (SB), i.e. the well-known sequences in the horse industry (e.g. weaving, cribbing), and “abnormal repetitive behaviours” (ARB) the sequences less described or recognized (*e*.*g*. [35; 69]). The 5 SB and the 9 ARB observed in this study as well as their prevalence are described in Table 1 (see results part).

**Table 1:** Description and prevalence of the SB and ARB observed in our study (from [25, 70]).

Type	Description	Prevalence N *(% individuals)*
**SB**	**- weaving:** lateral movement of head, neck, forequarters and sometimes hindquarters,	5 (*6%*)
	**- cribbing/windsucking:** the horse grasps a fixed object with its incisors, pulls backwards and draws air into its oesophagus,	6 (*7%*)
	**- head tossing/nodding:** vertical movements of head and neck,	9 (*11%*)
	**- stall walking:** repetitively tracing a route within the stall.	2 (*2%*)
**ARB**	**- repetitive licking:** licking of the same object in its environment (except the trough),	5 (*6%*)
	**- repetitive biting:** biting of the same object in its environment (except the trough),	4 (*5%*)
	**- head movements** (other than head tossing / nodding): movement of the head,	5 (*6%*)
	**- mouth open:** the horse keeps its mouth open with a lateral movement of its neck,	2 (*2%*)
	**- teeth rubbing:** rubbing teeth on the upper part of the door,	1 (*1%*)
	**- teeth chattering:** mouth movement with teeth chattering,	1 (*1%*)
	**- tongue movements**: movements of tongue, inside or outside the mouth	3 (*4%*)
**Total**		**34 (*41%*)**

As they share the same definition (repetitive and apparently functionless behaviours [70] and appear under sub-optimal conditions, SB and ARB were analysed together.

In the present study, the observer stood motionless at one end or in the middle of lines of stalls. In many cases, the stables were disposed along corridors with a row of stalls on each side. When positioned at the midline of the corridor, it was therefore easily possible to see 4 stalls at a time. When the stables were laid out so that the stalls had an opening to the outside, it was generally possible to see 6 horses in a row. The sampling was *ad libitum* [71] and the selected behaviours were scored (in terms of presence/absence) every time they occurred, together with the horse’s identity. For a behaviour to be considered as SB/ARB, the behavioural sequence had to be repeated at least 3 times successively and observed 5 times, independently of the period of observation. Most observations were performed at quiet times (outside teaching activities) with little disturbance by the routine procedures. In any case, the proportion of quiet and disturbed time periods was balanced between stables so that they remained comparable. In total, each horse was observed for 18h. At the end of the procedure, horses were scored on a binary basis: 1 if they performed at least one SB/ARB, 0 otherwise.

#### Depressed-like postures

In several situations, when welfare was altered, some animals were described as apathetic and showed a strong decrease of responses towards their environment [24; 72; 73]. **“Depressed-like” posture** in riding school horses was characterized for the first time by Fureix et al [25]: the animals stand immobile, eyes wide open with a the neck stretched (back and neck on the same line), a gaze, head and ears fixity, ears generally backwards, complete indifference towards environmental stimuli (visual, tactile and auditory [26; 74]) when displaying the posture, in their home environment, and with signs of anhedonia [24].

The prevalence of “depressed like posture” was evaluated at the same time as SB/ARB and following the exact same procedure. At the end of the observation time (18 h/horse, see above), horses were classified: 1 if at least once they showed the “depressed-like” posture” during the observation time, 0 otherwise.

#### Ears position

These positions are used as a welfare (and pain) indicator in several studies. It was defined by referring to studies on other species (*e*.*g*. [75]): axial ears (perpendicular to the head–rump axis), forward ears (tip of the ears towards the front at an angle of more than 30° from the perpendicular) or backward ears (tip of the ears towards the back at more than 30° from the perpendicular). In horses, backwards ears position is reported in cases of acute pain or discomfort (review in [73]), and has been shown to be associated with physiological disorders [76] and/or a negative perception of its living conditions [20].

Ear positions were recorded whilst horses were foraging on the ground (hay/straw) only, as it has been shown to be a reliable context (*e*.*g*. [20]). Observations were made when the stables were quiet, outside feeding and working time. The experimenter walked slowly and regularly (1step/s) in the middle of the corridor, or 2m away from the stalls in stables with one line of stalls. She approached each stall slowly in order to see the ear positions through the trough opening or door, remaining at a distance. This quiet approach did not elicit the strong reactions observed when approaching the door suddenly [77]. The instantaneous ear position of the feeding horse was silently noted (only if the horse kept feeding and paid no attention to the observer). The observer then resumed her walk along the midline up to the next stall. These samples were taken every day for 3 consecutive days and distributed throughout the day until 10 ear positions were obtained per horse. The percentage of scans in each position was calculated for each horse. For further analyses horses were categorized according to their “favourite” (≥60% of scans) posture: mostly forwards ears/mostly backwards ears. Asymetric or lateral positions were considered as “neutral” and were in any case observed less frequently (never as a predominant position).

### Postural measures (geometric morphometry)

#### Data recording

In order to locate anatomical points later in the photographs (future landmarks), seven marks (grey clay points, visible on all coat colours) were drawn on the horses on the side with less mane (then, if required, photographs were horizontally turned in order that they were all of the same orientation). The marks were placed in a sagittal plane in relation to skeletal cues (thus corresponding to anatomically homologous points) from head to croup along the spine. Marks were placed on: the first coccygeal vertebra; the lumbo-sacral and the thoraco-lumbar junctions; the tenth thoracic vertebra (corresponding to the lower point of the withers); the atlas; the temporo-mandibular joint; the tubercle of the facial crest [64].

**Fig 1:**
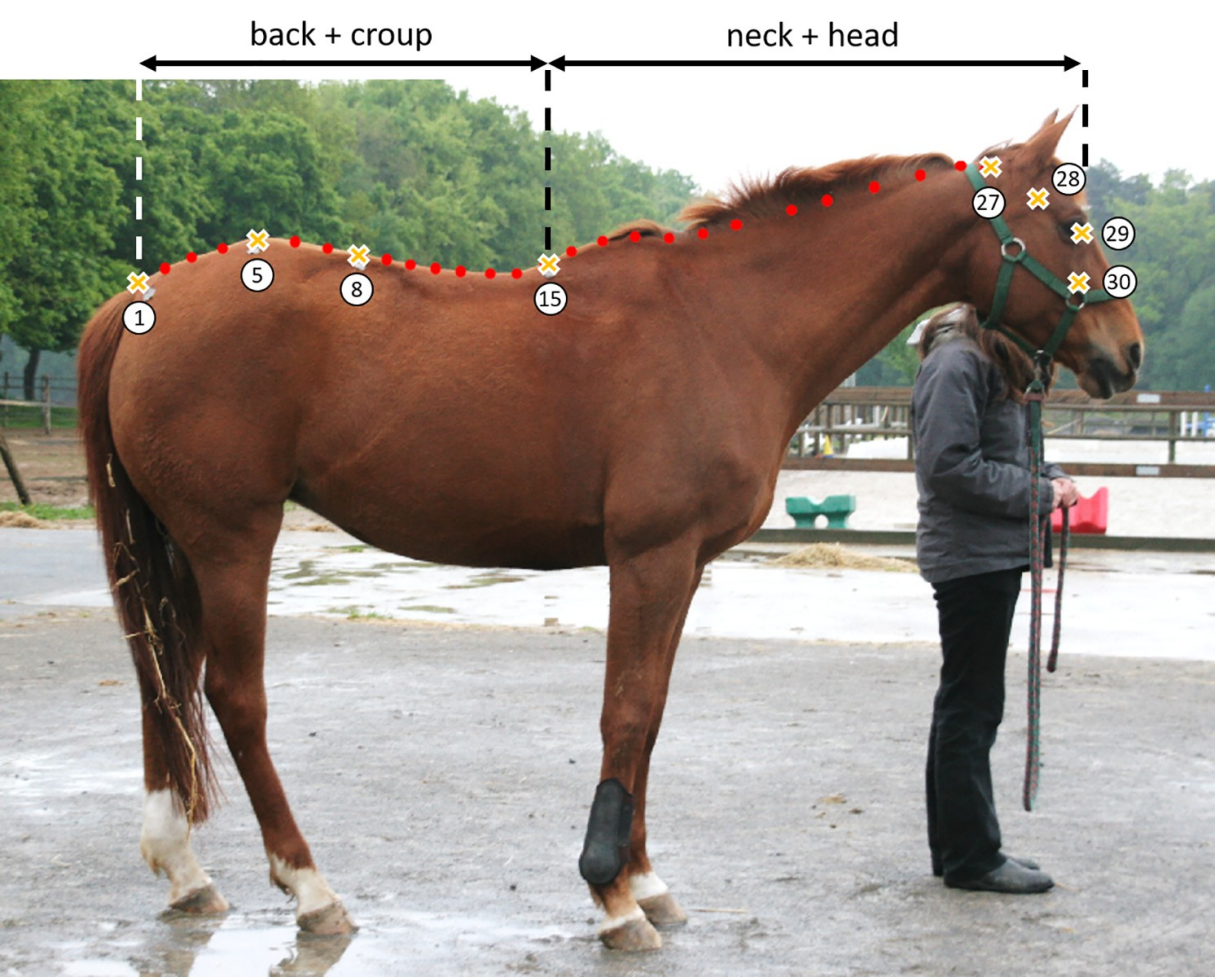
Location of the different points used for the mixed method. The landmarks are represented as crosses and the SSL as red points, and points used for the study of parts of the dorsal outline.

Horses were observed while being halter led by an unfamiliar experimenter, when walking and standing motionless (Fig 1). The experimenter did not talk to the horse, stayed on its left side and held the rope slackly at a predefined distance from the horse’s head (1m), so that the experimenter never pulled the rope or the horse’s head. Horses’ postures were recorded using photographs taken perpendicularly 10±1 m from the horse (digital camera Canon EOS 20D, zoom lens 50 mm to limit perspective distortions).

The previous study had shown that 10 photographs while standing motionless, and 20 photographs while walking led by the experimenter were necessary to account for intra-individual variability [64]. The median number of photographs per individuals while standing motionless was thus 10 (which is also equal to the first and third quartiles), with a range (minimum and maximum) of 6 to 12 respectively, whereas the median number of photographs per individual while walking led by an experimenter was 20 (as the first and third quartiles), with range (minimum and maximum) of 14 and 32 respectively.

#### Morphometric geometric treatment

The thirty landmarks (LD) were digitized for all horses by one single experimenter (E.S., previously trained to use this specific set of landmarks), from the photographs, using tpsDig2 software (tps software are available on http://life.bio.sunysb.edu/morph/). The files were then loaded from tpsDig2 into tpsUtil to be combined into a single file.

This file was then loaded into R (version 3.1.2, The R Foundation for Statistical Computing, http://www.r-project.org/foundation/) to create sliding semilandmarks (SSL) and to begin the shape analysis (R libraries: *ade4*, version 1.7–2; *geomorph*, version 2.1.6). SSL were defined according to the approach that minimizes the bending energy [78]. Generalized Procrustes Analysis [79] and Principal Component Analysis (PCA) were then conducted to visualize the distribution of the shape configurations corresponding to horse postures.

Based on the prior methodological study, the two most optimal geometric morphometric methods of shape analysis were used: 1) the mixed method using the 7 marks and the nasal corner of the eye as LD and the 22 other points drawn on the upper line of the horses are defined as SSL. 2) the Semi Sliding landmarks (SSL) method using just the medial canthus of the eye as LD and the 29 others points were defined as SSL (Fig 1).

The object of the mixed method is to draw a curve with SSL while keeping anatomical information thanks to the LD. The purpose of the SSL method is to limit as much as possible errors of LD positioning.

The movement of the neck (corresponding to a rotation around the withers) can be cancelled thanks to the R library *geomorph*. Therefore, we chose to stabilize the angle formed by the LD number 1, 15 and 30.

The two GM methods were applied to the dorsal profile of the horse, or just on sections of the spine in order to study if some portions are more informative than others. By deleting some LD or SSL, we could focus just on back and croup (points 1 to 15, Fig 1), or just on neck and head (points 15 to 30). The methodological study [64] had shown that:

- when studying the entire dorsal outline of the horse, the SSL method without rotation of the neck is the most appropriate,
- both the mixed and SSL methods are relevant when applied only on neck and head or on croup and back to discriminate riding schools. The reason for using these two methods is that they provide different deformation grids on the three first principal components. Therefore they are useful for identifying which precise element of the posture is associated with a welfare indicator.

### Statistical analysis

The effect of the different parameters was studied on the first three principal components (abbreviated PC) resulting from the Principal Component Analysis (PCA) based on the Procrustes coordinates from the GM methods [79] used thanks to mixed model Analyses of Variance (ANOVA), where individuals were considered as a random factor. The F-statistic values resulting from the ANOVAs were extracted to compare the effect of one parameter between the different methods, and the p-values to determine the impact of the parameters.

Associations between two quantitative variables were tested thanks to the Pearson’s correlation test, between one quantitative and one qualitative variable with the non-parametric Kruskal-Wallis test, and between two qualitative variables thanks to the Chi-square test (with the Monte Carlo method if one count was inferior to 5).

The statistical analyses and graphic illustrations were performed with R version 3.1.2, using the *geomorph* (version 2.1.6), *ade4* (version 1.7–2) and *lme4* (version 1.1.18) libraries. The level of significance of all the statistical tests was set at 5%.

## Results

### Horse population characteristics

The studied population represented 85 horses (50 geldings and 35 mares) of various ages (7–20y.o, median age = 14) and breeds (N = 9, mostly unregistered horses). The horses’ age and sex distribution did not differ between schools (respectively Kruskall-Wallis (KW), p = 0,075; and chi-squared, p = 0.524).There were almost as many ponies (< 1.48 m at withers, N = 43, 51%) as horses (> 1.48 m at withers, N = 42, 49%). Most animals were mesomorphic (length = height, N = 61, 72%), the others were either brachymorphic (length < height: N = 13 (15%)) or dolichomorphic (length > height N = 11 (13%)).

Strong differences appeared between schools, according to the management parameters recorded: The number of hay meals varied from 1 (N = 45, 52.9%) to 3 (N = 13, 15.3%) with 7 horses having ad libitum hay provision (Monte-Carlo chi-squared, p < 0.001)

Horses spent 0 to 95% of their time in paddock (KW U test, p < 0.001), either alone or in group (respectively N = 24 and N = 53; Monte-Carlo chi-squared, p < 0.001).

When is their stalls, they could see 0 to 11 conspecifics (KW, p < 0.001).

Depending on the riding schools, the time spent working per week varied from 3 to 14 hours per week (KW, p < 0.001).

Accordingly, large differences between schools were also observed in the welfare indicators recorded:

Depending on the riding school, 0 to 100% of the horses displayed SB/ARB (Monte-Carlo chi-squared, p = 0.002) (Table 1), 0 to 60% (N = 15, 18%) presented the depressed-like posture (Monte-Carlo chi-squared, p = 0.024), 0 to 90% (N = 31, 27%) had predominantly backwards ears whereas 0 to 75% had predominantly forward ears (Monte-Carlo chi-squared, p = 0.020). Some horses (N = 4, 5%) performed more than 1 SB/ARB.

### Postures associated with welfare indicators

The entire dorsal outline of the horses was first investigated to try to identify postures associated with the welfare indicators. The information provided by the neck and head or the back and croup were then examined.

#### Posture of the dorsal outline without neck movement (SSL and mixed method without neck rotationPostures and welfare indicators

The PCA of the SSL method showed that between 75.2 and 76.6% of the variance was explained by the three first components both for the SSL and the mixed method and whether the horse was standing motionless or walking (Table 2).

**Table 2:** Percentage of variance and ANOVA results of the first three principal components (PCs) of the landmarks configuration PCA of SSL and mixed method on the dorsum without neck rotation.

	Standing motionless F value and *p-value*			Hand walking F value and *p-value*		
	SSL method					
Principal component (% of variance of the axis)	PC1 (48.4%)	PC2 (15.4%)	PC3 (11.4%)	PC1 (42.6%)	PC2 (19.5%)	PC3 (14.5%)
SB/ARB	**5.25** ***0*.*022***	1.54 *0*.*215*	1.22 *0*.*27*	1.96 *0*.*161*	1.04 *0*.*308*	3.73 *0*.*053*
“Depressed-like” posture	0.26 *0*.*61*	1.35 *0*.*245*	0.107 *0*.*744*	0.273 *0*.*601*	0.568 *0*.*451*	0.226 *0*.*635*
Ear position	0.341 *0*.*711*	2.8 *0*.*0607*	0.337 *0*.*714*	1.55 *0*.*212*	1.21 *0*.*298*	0.507 *0*.*603*
	Mixed method					
Principal component (% of variance of the axis)	PC1 (46.3%)	PC2 (16.1%)	PC3 (11.1%)	PC1 (41.9%)	PC2 (21%)	PC3 (11.1%)
SB/ARB	**4.75** ***0*.*029***	1.87 *0*.*171*	2.74 *0*.*098*	1.96 *0*.*161*	1.04 *0*.*308*	3.73 *0*.*053*
“Depressed-like” posture	0.12 *0*.*734*	0.95 *0*.*329*	3.73 *0*.*053*	0.273 *0*.*601*	0.568 *0*.*451*	0.226 *0*.*635*
Ear position	0.341 *0*.*711*	2.8 *0*.*0607*	0.337 *0*.*714*	1.55 *0*.*212*	1.21 *0*.*298*	0.507 *0*.*603*

The ANOVA on the welfare indicators showed similar results for the two methods when standing motionless (Table 2) and the deformation grids corresponding to PC1s associated with welfare indicators were very similar in the mixed and SSL methods. Therefore we decided to focus on the SSL method when standing motionless, because of the higher variance of the axis. When hand walking, ANOVA results were not significant, thus we concentrated further only on the results obtained when the horse was standing motionless.

**Fig 2:**
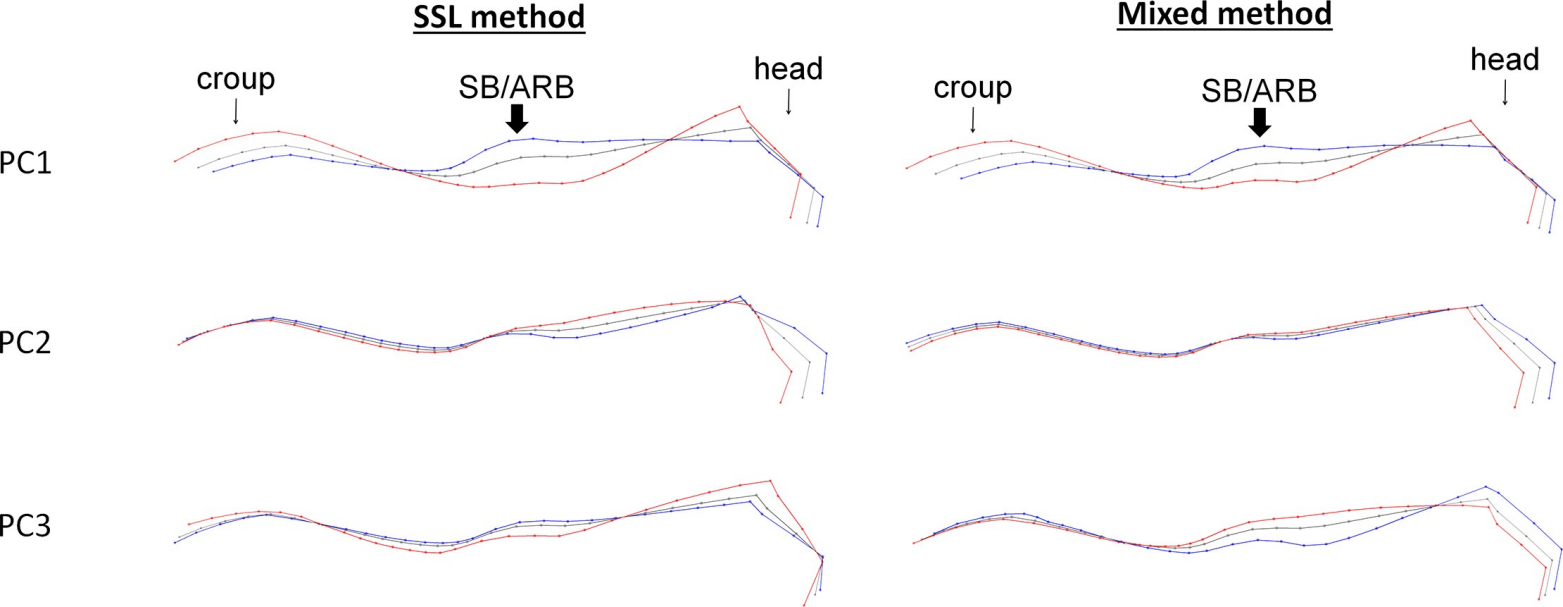
Deformation grids corresponding to the three first principal components of the SSL and mixed method on the dorsum without neck rotation, when standing motionless. Grey = consensus, red = minimum of the axis, blue = maximum of the axis. Arrows show the part of PC which includes the more individuals expressing SB/ARB (when result of the ANOVA is statistically significant).

When the horse was standing, the PC1 of the SSL method corresponded mainly to the withers shape, the relative size of the croup compared to the back, of the head compared to the neck, and to a lesser extent to the angle formed by head and neck (Fig 2). PC2 was supported by variations of the angle formed by head and neck and variations in neck shape (a round neck was associated with a narrower angle between head and neck and a hollow neck with an open head- neck angle). Finally, PC3 was associated with the relative size of the head compared to the neck, the head/neck angle and the way of holding the neck (open angle of the head/neck is associated with a relatively small head and low neck, whereas a narrow angle corresponds to a relatively large head and high neck) (Fig 2).

There was a clear link between postural morphometric measurements and the frequency of SB/ARB performed by the horses, with a significant effect on PC1 but none with the other welfare indicators measured.

**Fig 3:**
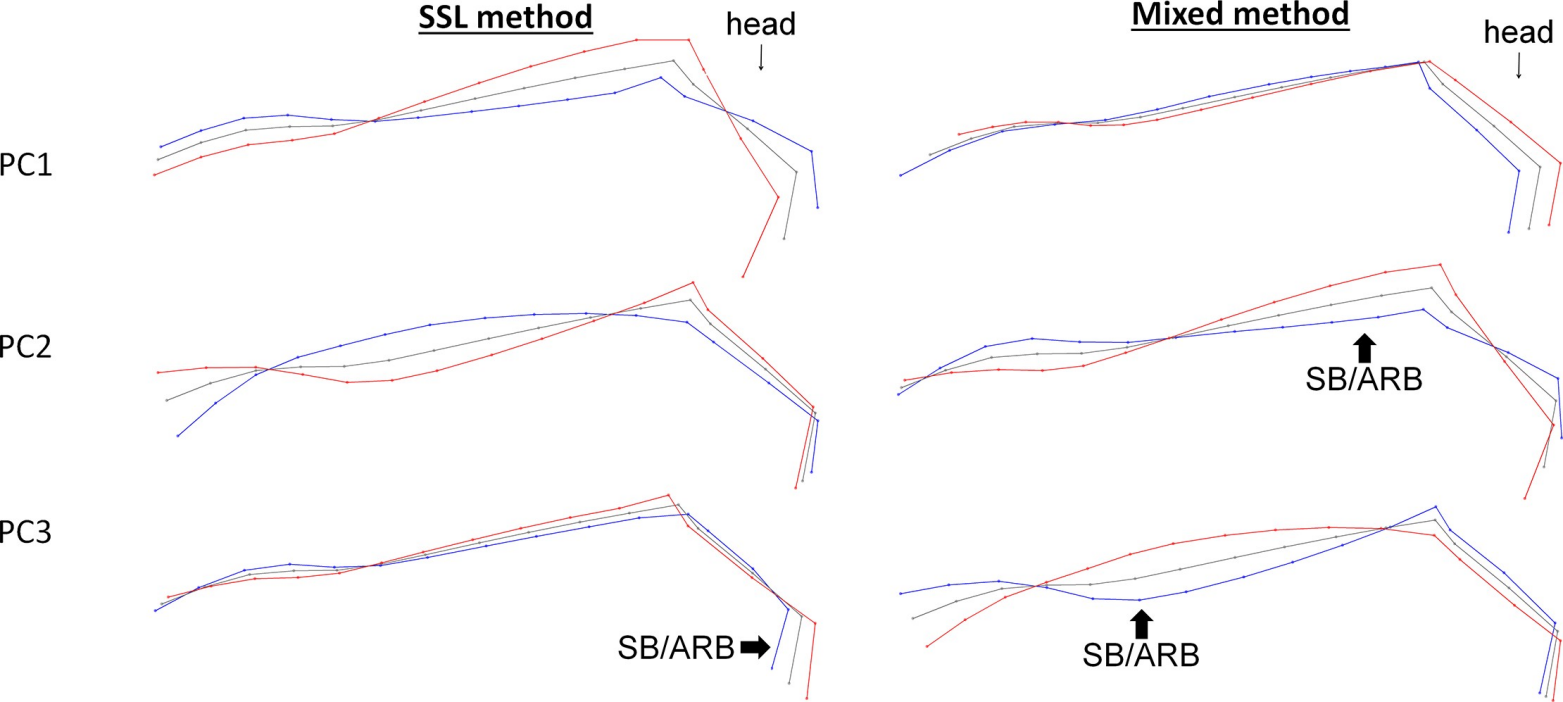
Deformation grids corresponding to the three first principal components of the SSL and mixed method on the head and neck when standing motionless. Grey = consensus, red = minimum of the axis, blue = maximum of the axis.

Horses performing SB/ARB in stall had more prominent withers, shorter croup and head compared to the rest of the body and the angle formed by head and neck was fairly open (flat/hollow neck) (Fig 3). Further analyses revealed that there was no difference on the PCs data according to the type of SB/ARB performed (oral vs motor) nor between locomotor (box walking) and head-related SB/ARB (ANOVAs, p > 0.05).

**Factors of influence**

PC1 was neither affected by age (ANOVA, p = 0.5) nor by sex (p = 0.683), but was associated with the type of equid (p < 0.001), the time spent working per week (p = 0.025), and the number of hay meals (p = 0.002) (Table 3). However, since the occurrence of SB/ARB was also associated with these factors [42], this may be a mere co-occurrence.

**Table 3:** Significant ANOVA results of the three first principal components (PC) of the landmarks configuration PCA of SSL and mixed method, and morphological/management indicators (F et *p-value*).

GM method		Type of equid	Proportions	Time spent in paddock	Time spent working	Number of meals of roughage	Number of visible conspecifics	Alone or in group in paddock
		Standing motionless						
PC1 SSL method on the dorsum without neck movement	F	27.70	4.42	0.46	5.05	4.90	0.13	0.23
	*p-value*	*< 0*.*001*	*0*.*012*	*0*.*499*	*0*.*025*	*0*.*002*	*0*.*910*	*0*.*628*
PC1 mixed method on the dorsum without neck movement	F	30.90	3.39	0.51	2.48	6.82	0.002	0.41
	*p-value*	*< 0*.*001*	*0*.*038*	*0*.*474*	*0*.*115*	*< 0*.*001*	*0*.*960*	*0*.*841*
PC2 mixed method on the head and neck	F	22.10	5.67	0.06	9.32	3.24	0.74	0.11
	*p-value*	*< 0*.*001*	*0*.*003*	*0*.*813*	*0*.*002*	*0*.*021*	*0*.*388*	*0*.*915*
PC2 mixed method on the back and croup	F	0.06	1.61	0.09	16.40	4.37	0.515	1.65
	*p-value*	*0*.*801*	*0*.*199*	*0*.*769*	*< 0*.*001*	*0*.*004*	*0*.*473*	*0*.*199*
PC3 SSL method on the head and neck	F	19.60	2.59	0.30	7.18	1.57	0.88	0.46
	*p-value*	*< 0*.*001*	*0*.*075*	*0*.*586*	*0*.*007*	*0*.*195*	*0*.*348*	*0*.*497*
PC3 mixed method on the head and neck	F	0.003	2.17	6.05	2.46	1.44	0.96	7.57
	*p-value*	*0*.*956*	*0*.*114*	*0*.*014*	*0*.*117*	*0*.*229*	*0*.*326*	*0*.*005*

Since SB/ARB horses seemed to differ in several postural aspects such as the subtle combination of relative sizes of body parts, the roundness of the neck and the associated head/neck angle, we examined more closely some details of the back.

#### Neck and head posture

**Postures and welfare indicators**

When concentrating only on the neck and head, it appeared that the three first components explained between 79.9 and 87.1% of the variance for both methods and situations (Table 4).

**Table 4:** Percentage of variance and ANOVA results of the first three principal components (PCs) of the landmarks configuration PCA of SSL and mixed method on the head and neck.

		SSL method			Mixed method		
		Standing motionless					
Principal component (% of variance of the axis)		PC1 (49.7%)	PC2 (22.4%)	PC3 (15%)	PC1 (39.8%)	PC2 (23.9%)	PC3 (16.2%)
SB/ARB	F *p-value*	1.49 *0*.*222*	2.11 *0*.*146*	**14.3** ***<0*.*001***	1.27 *0*.*26*	**4.87** ***0*.*027***	**58** ***0*.*016***
“Depressed-like” posture	F *p-value*	0.065 *0*.*799*	2.26 *0*.*133*	0.088 *0*.*767*	1.15 *0*.*284*	0.031 *0*.*859*	3.05 *0*.*081*
Ears position	F *p-value*	0.364 *0*.*695*	2.27 *0*.*104*	2.65 *0*.*071*	0.030 *0*.*971*	0.192 *0*.*825*	2.13 *0*.*119*
		Walking led by an experimenter					
Principal component (% of variance of the axis)		PC1 (46.5%)	PC2 (27.7%)	PC3 (12.9%)	PC1 (32.7%)	PC2 (24.4%)	PC3 (20.2%)
SB/ARB	F *p-value*	1.16 *0*.*281*	**3.94** ***0*.*047***	**8.56** ***0*.*003***	3.27 *0*.*070*	2.46 *0*.*117*	**7.49** ***0*.*006***
“Depressed-like” posture	F *p-value*	< 0.001 *0*.*989*	0.88 *0*.*348*	0.482 *0*.*487*	0.22 *0*.*639*	1.81 *0*.*179*	0.0133 *0*.*908*
Ears position	F *p-value*	0.909 *0*.*403*	0.072 *0*.*93*	2.08 *0*.*125*	1.46 *0*.*232*	0.606 *0*.*545*	2.5 *0*.*0821*

**Fig 4:**
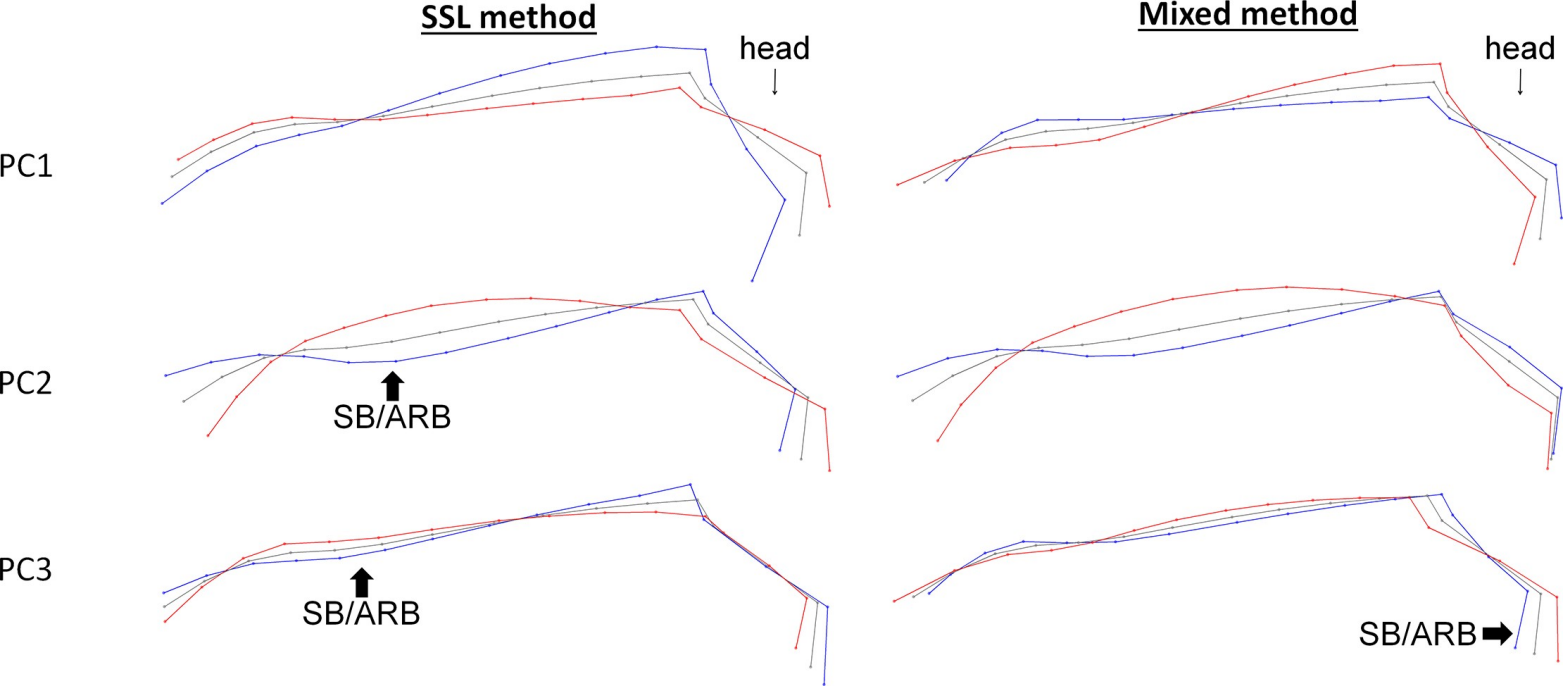
Deformation grids corresponding to the three first principal components of the SSL and mixed method on the head and neck when hand walking. Grey = consensus, red = minimum of the axis, blue = maximum of the axis).

Figs 3 and 4 show the associated deformation grids. Both methods and situations showed the same results as those obtained with the entire back: a significant relationship was found with the occurrence of SB/ARB, but none with the other welfare indicators (Table 4).

Thus, the presence of SB/ARB was associated with (Fig 3):

- withers shape and the relative size of the head compared to the neck: The individuals expressing SB/ARB had a relatively smaller head and more prominent withers than the others,
- the same elements in addition to the head/neck angle and/or the way of holding the neck: the individuals expressing SB/ARB showed a more open head/neck angle and/or a lower neck,
- the relative size of the head combined with the roundness and the way of holding the neck: individuals expressing SB/ARB had a relatively small head

The relative size of the head compared to the rest of the body seemed to be the essential element associated with the expression of SB/ARB. This observation is in accordance with the fact that individuals with “horse” type exhibited more SB/ARB (chi squared, p = 0.012) and had relatively small heads (e.g. standing motionless: ANOVA on the type of equids for PC3 of the SSL method and the PC2 of the mixed method show p < 0.001, p-value not significant for PC3 of the mixed method) (Table 3). As for the study of the entire back, we found no posture difference between oral and motor SB/AB and between boxwalkers and horses with other head related SB/ARB, neither when standing motionless nor when hand walking (ANOVAs, p > 0.05).

Overall thus, whether standing motionless or walking, hoses that exhibited SB/ARB in stall had characteristic postures such as a flat or hollow and high neck (open head-neck angle), a prominent wither but they were also characterized by a relatively smaller head compared to body size (horse type).

**Factors of influence**

When considering associated management or individual factors, the PC2 and PC3 of the SSL and mixed methods for standing motionless were associated (but not correlated, Pearson, r = 0.24, NS) with the time spent working per week (ANOVA, p = 0.007), i.e. alterations of the postures were not due to too many hours working but may have been related to work (quality?). When hand walked, the PCs of the two methods associated with the presence of SB/ARB were only related to the type of equid for the two PC3 (SSL method, p < 0.001; mixed method, p = 0.011).

#### Back and croup posture

**Postures and welfare indicators**

**Fig 5:**
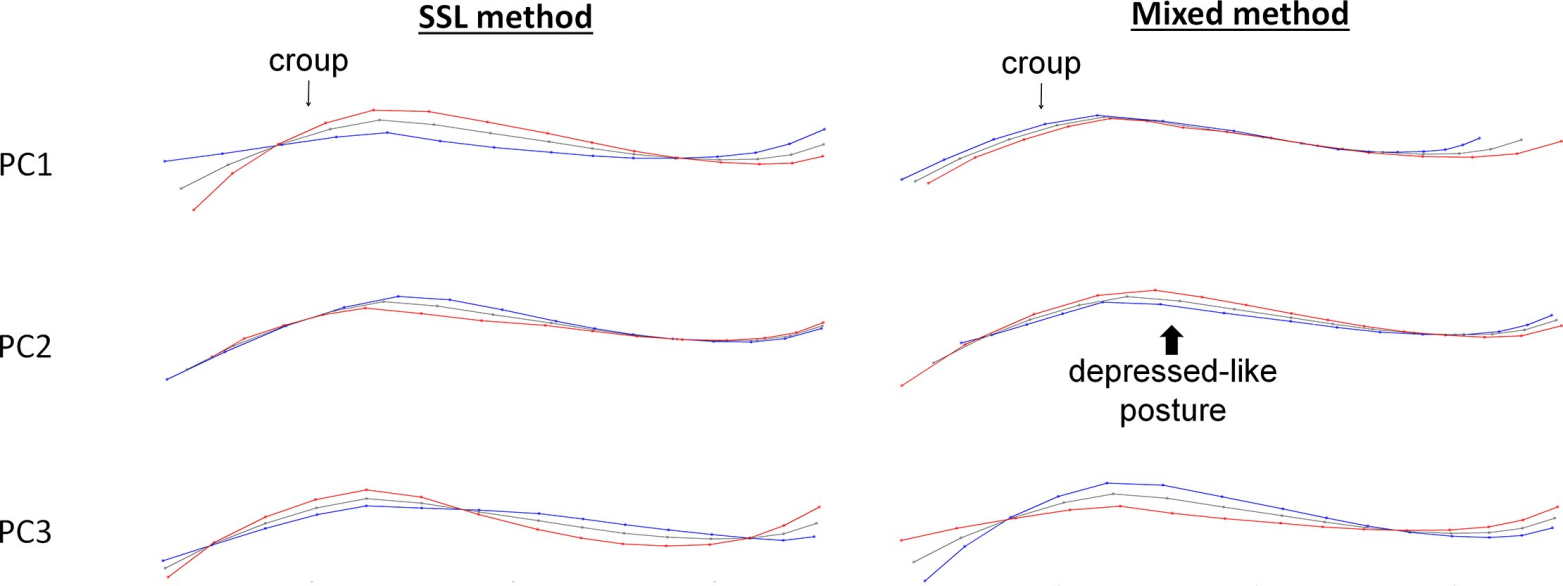
Deformation grids corresponding to the three first principal components of the SSL and mixed method on the croup and back when standing motionless. Grey = consensus, blue = minimum of the axis, red = maximum of the axis).

**Fig 6:**
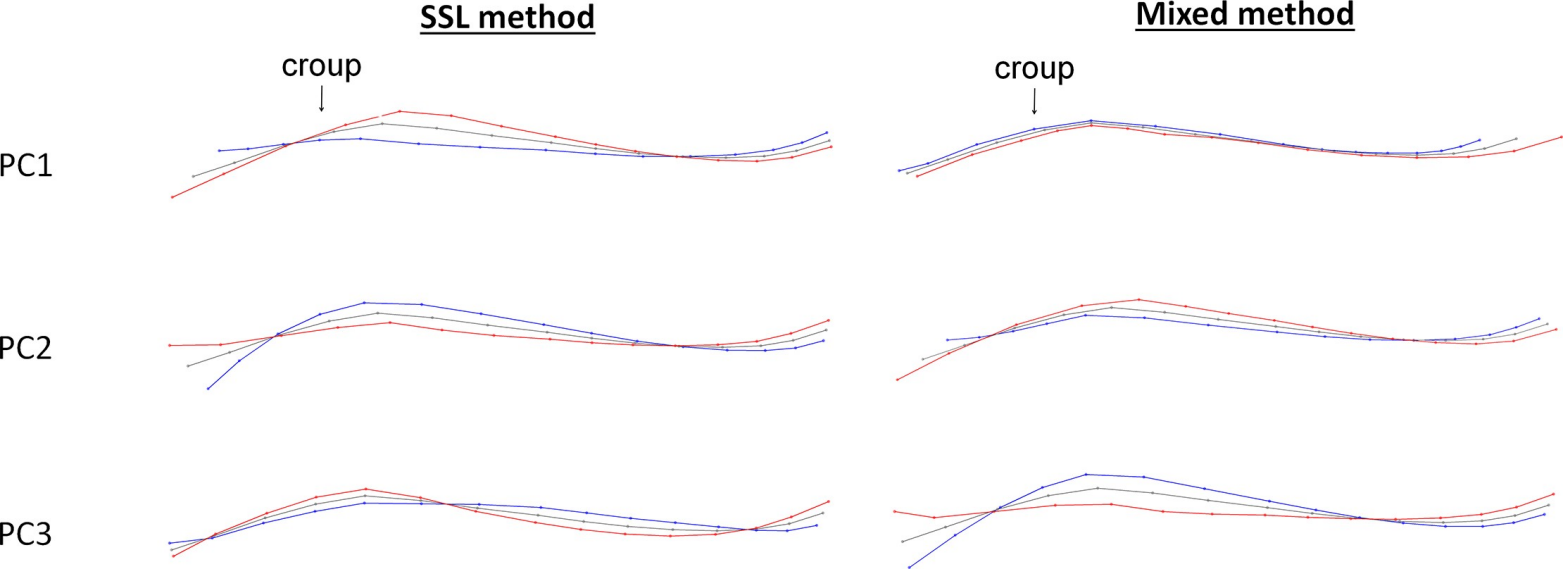
Deformation grids corresponding to the three first principal components of the SSL and mixed method on the croup and back when hand walking. Grey = consensus, blue = minimum of the axis, red = maximum of the axis.

Table 5 summarizes the findings obtained for both the SSL and mixed methods when horses were standing motionless or walking led. The three first components explained between 83 and 95% of the variance. Figs 5 and 6 show the associated deformation grids.

**Table 5:** Percentage of variance and ANOVA results of the first three principal components (PC) of the landmarks configuration PCA of SSL and mixed method on the back and croup. PC4 was discarded because of a percentage of variability < 10%.

	SSL method F value and *p-value*			Mixed method F value and *p-value*		
	Standing motionless					
Principal component (% of variance of the axis)	PC1 (39.3%)	PC2 (35.8%)	PC3 (11.9%)	PC1 (57.8%)	PC2 (24.4%)	PC3 (10.7%)
SB/ARB	2,11 *0*.*146*	2,08 *0*.*15*	2,92 *0*.*087*	0,763 *0*.*382*	2,25 *0*.*134*	1,87 *0*.*171*
“Depressed-like” posture	0.619 *0*.*432*	0.006 *0*.*938*	0.011 *0*.*917*	0.548 *0*.*459*	**3.85** ***0*.*050***	0.08 *0*.*778*
Ears position	2.34 *0*.*096*	0.733 *0*.*481*	0.323 *0*.*724*	1.37 *0*.*253*	0.953 *0*.*385*	1.94 *0*.*144*
	Walking led by an experimenter					
Principal component (% of variance of the axis)	PC1 (43.3%)	PC2 (34.7%)	PC3 (10.9%)	PC1 (55.3%)	PC2 (23.9%)	PC3 (17.8%)
SB/ARB	0.617 *0*.*432*	**4.09** ***0*.*043***	0.547 *0*.*459*	0.121 *0*.*728*	1.52 *0*.*217*	2.33 *0*.*127*
“Depressed-like” posture	0.002 *0*.*989*	0.423 *0*.*516*	0.101 *0*.*75*	0.126 *0*.*722*	**5.48** ***0*.*019***	0.554 *0*.*457*
Ears position	1.12 *0*.*326*	0.557 *0*.*573*	0.364 *0*.*695*	0.504 *0*.*604*	0.906 *0*.*404*	2.68 *0*.*068*

When standing motionless and hand walking, the PC2 of the mixed method was associated with **“**depressed-like” posture while no relation could be found between back and croup shapes, the expression of SB/ARB or the ear positions. The deformation grids associated with PC2 correspond to a combination of the form/roundness of the croup and the relative size of the croup compared to the back. Horses showing “depressed-like” posture had more often a relative small, flat or hollow croup. Other PCs of the two methods were related to either just the relative size of the croup or its shape. Therefore, the combination of the two elements of posture (size and shape of the croup) was characteristic of horses with “depressed-like” postures.

However, the PC2 of the SSL method when the horse was walking was also related to the presence of SB/ARB. Deformation grids include a difference of roundness of the croup and back, and a different way of holding the tail. Horses expressing SB/ARB hold their tail higher, had a flat or hollow croup, and back. As for the study of the head and neck, we found once again no posture difference between oral and motor SB/AB and between the boxwalker and the horses with other head related SB/ARB, whether the horse was standing motionless or hand walked (ANOVAs, p > 0.05).

To summarize when standing motionless, a **relatively small flat or hollow croup** was associated with a “depressed-like” posture while when walking these same characteristics were associated with both depressed postures and SB/ARB expressed when in stall.

**Factors of influence**

Further analyses confirmed that there was no association between the type of equid or its proportion, and the occurrence of “depressed-like” postures (for the type of equid: chi-squared, p = 0.277; for the proportion: Monte-Carlo chi-squared: p = 0.283), which means that this does not explain the association between the size or shape of the croup and the depressed states. In fact PC2 of the mixed method was also associated with the time spent working per week (ANOVA, p < 0.001). Moreover, horses that expressed “depressed-like” posture were characterized by the time they spent working per week (Kruskal-Wallis, p = 0.009) but were not those which worked the most: 11 of the 15 horses expressing “depressed-like” posture worked between 6 and 8 hours per week (maximum of the sample: 14 hours per week).

The number of hay meals was also related to PC2 of the mixed method (ANOVA, p = 0.004). Horses showing “depressed-like” posture had fewer hay meals (only one or two meals) (chi-squared, p = 0.002).

However, the PC2 of the mixed method was more related to the time spent working per week (ANOVA, p < 0.001) than to the number of hay meals (ANOVA, p = 0.004), suggesting a higher influence of work than food on the croup and back shapes.

When studying the back and croup, the relative size of the croup compared to the back, the PC2 of the SSL method was not associated with the type of equid (pony/horse: ANOVA, p = 0.109) (Table 3). However this PC was strongly related to the horse’s proportion (ANOVA, p < 0.001) and the number of hay meals (ANOVA, p = 0.004). As mentioned earlier for the study of the head and neck posture, individuals expressing SB/ARB have more dolichol / mesomorphic proportions and fewer hay meals.

**Fig 7:**
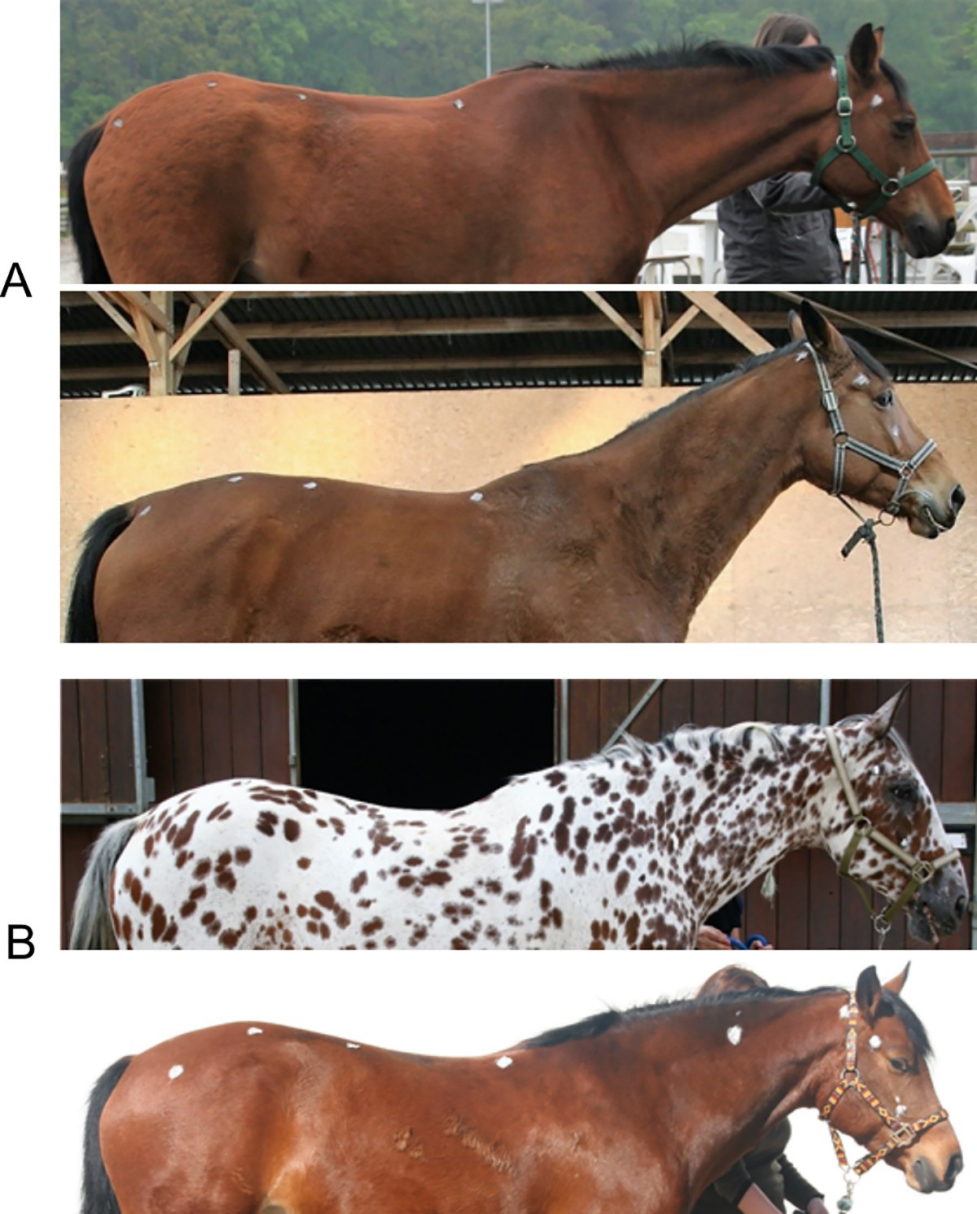
Photographs of two types of riding school horses. (A) Photographs of riding school horses presenting characteristic elements of posture associated with poor welfare. They all express SB/ARB and show “depressed-like” posture too. (B) Photographs of riding school horses which don’t express SB/ARB and “depressed-like” posture and without characteristic elements of posture associated with poor welfare.

Thus, animals with an impaired welfare state expressed through SB/ARB and depressed-like syndrome can clearly be differentiated through their overall posture (Fig 7)

## Discussion

The application of 2 different methods of geometric morphometrics in two situations (horses standing motionless or walk led) on the shape of the whole dorsum or parts of it reveals surprisingly consistent results: overall stereotypic or depressed horses living in restricted conditions are characterized by a flat or hollow back, neck and croup shape with a prominent withers. These findings show that a poor welfare is also associated with chronic alterations of posture, erasing the natural curves of the horse’s spine. These results confirm and deepen the results obtained in earlier studies [24; 26].

This study on horses with different welfare states confirms the interest of geometric morphometrics and the development of dedicated approaches [64]. One of the GM's strengths is to be able to identify some subtle shape variations, and indicate the pertinent elements of posture. The identified dorsal profile constituted a signal which, when combined with other welfare indicators, may indicate some welfare alteration. However, studies on posture in other species have found that pigs reared in social isolation had a rounder back than those reared socially [43]. Extreme arched back postures are also found in case of acute pain in horses (*e*.*g*. [47–49]) and other species (sheep [42–43], cattle [44–45] and pig [46]). In these cases, the arched posture is generated by the contraction of the abdominal muscles and the flexion of the vertebral column with possibly some degree of dorsally located back muscle stretching. This posture may reduce the weight on the limb affected by the lameness or laminitis. Therefore, postural indicators of chronic stress may differ from those of acute pain for a same species.

The profiles found here also resemble those described for horses experiencing chronic back problems that show a flat or hollow neck [22], a head held high [80] and flat and rigid backs [40–53] (review in [39]). We had no assessment of the spine state for these horses, but the relationship found between the profile and the time spent working (although it was not linear) may suggest that in these riding schools as in those previously investigated, inappropriate riding techniques or tackle may be a source of back problems and emergence of stereotypies and depressive states [37–39]. It was found that riding school horses have particular profiles compared to recreational horses [25] and therefore, the association found here between back postures and indicators of welfare might be different in horses used in other types of work. Only further studies will allow to respond to this question [64].

Beyond this potential influence of work and back pain, these postural profiles were associated with the horse’s characteristics (*i*.*e*. type of equid and proportions) and management factors (*i*.*e*. number of hay meals, time spent working and at the paddock, being released alone or as a group in paddock). In accordance with Lesimple et al. [38], in this subset of horses, the number of hay meals, the time spent in paddock, and whether horses were released alone or in group in paddock appeared important factors for the emergence of SB/ARB, but also for the associated postures. The morphology of the horse can modulate the effects of working and housing conditions: horses with shorter backs (mostly of the pony type and with brachymorphic proportions) are less prone to injuries of soft tissues than horses with long backs (mostly of the horse type and with dolicho/mesomorphic proportions) [49]. Lesimple et al [38] showed that horses were more at risk of developing SB/ARB as a result of riding techniques than ponies. The fact that the studied population presented a large morphology panel added complexity but despite of that, postural profiles associated with welfare indicators clearly stood out.

Depressed horses were characterized by a flat croup which may either be due to the repetition of “depressed-like” postures where the horse puts most weight on the forelegs, hence lowering muscle activity at the croup level. It may be also that dorsal problems at the croup level induce depressive state in the horse. The results suggest that the depressed state, associated with flat croup and back, may be more related to work quality. The absence of clear relationship between postures and chronic ear positions further suggests that postural alteration are due to specific problems, while repeated backwards ears reflect a more general impaired welfare state [20;38].

Biomechanics of the equine spine can help understanding the present findings. According to the “bow and string” theory (Strasser 1913, in Jeffcott [49]), the trunk of the skeleton (*i*.*e*. the vertebral column, the pelvis and their muscles) works like a bow kept under tension from a string formed by the sternum, the abdominal muscles and the *linea alba*. The movements of the fore and hind legs bend or stretch the bow. Biomechanically, the head, neck, back and fore and hindquarters are connected. All actions that produce an elevation of the head, a decrease of the protractors of the forelegs and of the retractors of the hindlegs, or a contraction of the epaxial musculature of back lead to an extension of the back, which could become hollow. Such modifications may be induced by different factors:

Looking at the riding technics and equipment effects, several studies showed that inexperienced riders have inappropriate balance and hand actions that induce alterations of the horse’s back health (see [39] for a review). For example, Lesimple et al. [37, 39] have observed that the extent of vertebral problems of riding school horse was correlated with postures at work (neck height and shape), and in turn were clearly correlated with (and determined by) the riders’ hand positions Back disorders are known to affect the kinematics of the back by stiffening it [58]. Ridgway and Harman [40] commented that “back muscles pain produces a poorly developed top line, atrophy of the top line, weak muscling through the loins, prominent tuber sacrale, “swayback” or lordosis, prominent withers with poor muscling, poor gluteal muscle development, uneven shoulders, and often poor neck muscle development with more ventral neck muscle development”. This picture corresponds to a typical posture of a horse with compromised welfare.

- The use of inappropriate riding equipment, such as a poorly fitted saddle or bit, or tight noseband, causes or aggravates behavioural problems, discomfort and back disorders [39; 41; 54–55].
- The emotional state: In humans, it is well established that the normal posture can be altered by repetitive muscular or musculoskeletal tensions, caused by chronic emotions [81–82] or imposed working postures (e.g. children in classroom [83], computer and office workers. In horses, poor welfare can be associated with higher emotional states [28]. One can think that this may influence their chronic postures as is also the case in pigs [19].

In conclusion, the finding, based on precise geometric morphometric measurements, that a riding school horse’s flat or hollow dorsal profile is related to a compromised welfare state and associated management conditions provides a first clear profile of individuals with risk factors of at least a“depressed” or “abnormal” psychological state. These profiles may however be related to the particular population considered (riding school horses). Further studies will have to consider a larger panel of populations (*e*.*g*. other types of work) and to assess the spine state [64].
